# Sevoflurane Sedation for High-Risk Airway Management in the Pediatric Intensive Care Unit Following Sclerotherapy for a Pediatric Cervical Lymphatic Malformation: A Case Report

**DOI:** 10.7759/cureus.104588

**Published:** 2026-03-02

**Authors:** Kyosuke Sasaki, Aya Kajihama, Kaori Yamada, Hiroyuki Shimizu, Takuya Hayashi

**Affiliations:** 1 Department of Pediatrics, Yokohama Ryoiku Medical Center, Yokohama, JPN; 2 Department of Emergency and Critical Care Medicine, Kanagawa Children's Medical Center, Yokohama, JPN

**Keywords:** airway obstruction, bleomycin sclerotherapy, difficult airway management, inhalational sedation, lymphatic malformation, pediatric intensive care, postoperative sedation, rescue sedation, sedaconda, sevoflurane

## Abstract

Cervical lymphatic malformations (CLMs) can cause critical airway compromise due to post-sclerotherapy swelling, requiring precise sedation management in the pediatric intensive care unit (PICU). Precise airway management is especially challenging during the postoperative edema peak, where accidental extubation must be avoided. Inhalational sevoflurane provides rapid titration and predictable emergence, potentially allowing smoother management compared with traditional intravenous sedatives. We report the case of a one-year-old female patient (11.5 kg) with a massive CLM extending into the mediastinum who underwent bleomycin sclerotherapy. After the first procedure, intravenous sedation alone was insufficient, leading to agitation and unstable hemodynamics requiring vasopressor support. During the second procedure, similar agitation occurred despite initial intravenous sedation. Sevoflurane inhalational sedation via the Sedaconda® system was introduced 20 hours after PICU admission to achieve a stable and controllable sedation depth. This approach stabilized the patient, maintaining a State Behavioral Scale (SBS) of -1 to -2 at an end-tidal sevoflurane (EtSevo) concentration of 0.4-0.6%. It significantly reduced the requirement for intravenous agents and enabled smooth, predictable extubation without complications. Sevoflurane inhalational sedation may be a valuable alternative for sedation in pediatric patients at high risk for airway complications.

## Introduction

Cervical lymphatic malformations (CLMs) may cause significant airway distortion and postoperative swelling, requiring meticulous sedation management in the pediatric intensive care unit (PICU). Standard intravenous sedatives such as midazolam or fentanyl can be unpredictable due to pharmacological tolerance or paradoxical agitation, which increases the risk of accidental extubation. Inhalational sevoflurane offers rapid titration and measurable sedation depth via end-tidal concentration (EtSevo), potentially enabling smoother control. While the use of the Sedaconda® system (formerly AnaConDa®) has expanded in adult critical care, reports of inhaled sedation in the PICU remain limited [1,2], with emerging evidence primarily supporting its feasibility in older populations [3,4]. To our knowledge, this is the first documented case of Sedaconda® use for rescue sedation in a pediatric patient with cervical lymphatic malformation and high-risk airway following bleomycin sclerotherapy. We present a case in which rescue sevoflurane sedation enabled smooth airway management when multiple intravenous agents proved insufficient.

## Case presentation

**Figure 1:**
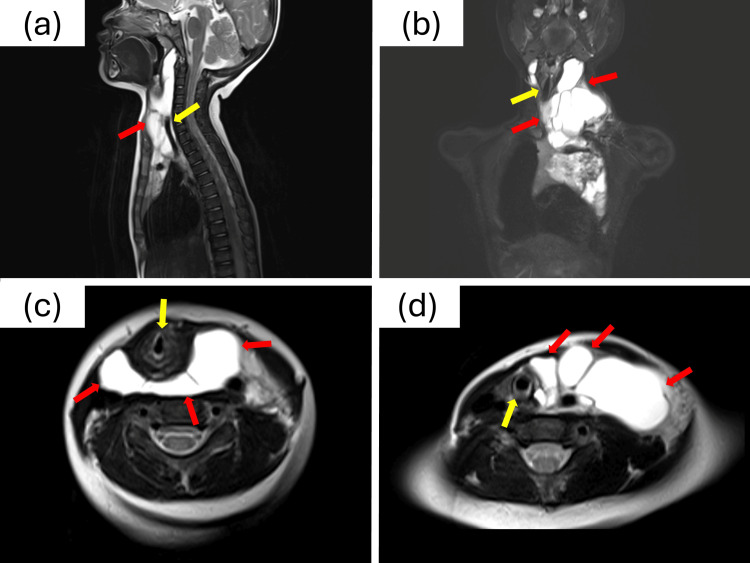
MRI of the multiloculated cervical lymphatic malformation (a) Sagittal T2-weighted image showing the lesion (red arrow) causing significant posterior displacement of the airway from the oropharynx to the trachea (yellow arrow). (b) Coronal T2-weighted image demonstrating the mediastinal extension (red arrows) and rightward deviation of the cervical trachea from the midline (yellow arrow). (c) Axial T2-weighted image at the oropharyngeal level, revealing the lymphatic malformation (red arrows) causing severe airway compression (yellow arrow). (d) Axial T2-weighted image at a lower level, showing circumferential compression of the tracheal lumen (yellow arrow) by the lesion (red arrows).

A one-year-seven-month-old female patient (11.5 kg) with no significant medical history was diagnosed with a large, multiloculated CLM extending from the oropharyngeal region to the upper mediastinum (Figure 1). Following the initial presentation, she underwent staged bleomycin sclerotherapy, with the first procedure at age one year eight months and the second, the index case of this report, at one year nine months.

First sclerotherapy course

Following the first procedure, the patient required six days of postoperative intubation due to anticipated airway swelling. Sedation was managed with a complex regimen of intravenous (IV) and enteral agents, including midazolam, fentanyl, dexmedetomidine, phenobarbital, triclofos sodium, tizanidine, and quetiapine. Despite these measures, the patient exhibited significant agitation and repetitive head movements, necessitating frequent boluses and resulting in unstable sedation depth. Furthermore, high doses of IV and enteral sedatives led to hemodynamic depression, requiring norepinephrine (up to 0.07 μg/kg/min) to maintain blood pressure.

Second sclerotherapy course

For the second sclerotherapy, the patient was admitted to the PICU intubated as planned. The patient was intubated with a 4.0 mm cuffed endotracheal tube, and arterial line monitoring was established for continuous blood pressure measurement. Similar to the first course, initial IV sedation with midazolam and fentanyl failed to control her agitation; persistent head movement raised critical concerns regarding accidental extubation during the peak edema period.

Given the failure of IV sedation and the complications during the previous course, sevoflurane rescue sedation was initiated 20 hours after PICU admission via the Sedaconda® system to achieve a more reproducible and controllable sedation depth (Table 1). EtSevo was titrated to 0.4-0.6%, achieving a stable State Behavioral Scale (SBS) [5], a validated and freely available sedation assessment tool, of -1 to -2.

**Table 1:** Sedation summary

Parameter	Observation
Age / Sex / Weight / Airway	1 year 9 months / female / 11.5 kg / 4.0mm cuffed ETT, 14cm oral
Diagnosis	Cervical lymphatic malformation (neck to mediastinum)
Procedure	Second bleomycin sclerotherapy
Sedation method	Sevoflurane with EtSevo monitoring
Duration of inhalational sedation	≈ 23 h
EtSevo (maintenance)	0.4–0.6%
EtSevo (pre-stop to pre-extubation)	0.8% to not detected
Extubation timing	Approximately 6 minutes after discontinuation
Adjunct agents	Fentanyl taper, midazolam tapered/ceased, and dexamethasone
Complications	None

This transition allowed for the discontinuation of midazolam and a reduction of the fentanyl infusion rate by more than half (from 0.9 to 0.4 μg/kg/h). Notably, hemodynamic stability was maintained without the need for norepinephrine, contrasting with the first treatment course. The patient remained stable (SpO₂ 98-100% on FiO₂ 0.21-0.23) throughout the duration of inhalational sedation (approximately 23 hours).

Airway evaluation and extubation

Extubation readiness was determined through a multidisciplinary approach. The surgical team performed fiberoptic laryngoscopic assessments to evaluate airway patency, while the PICU team confirmed physiologic readiness via leak tests (12 cmH₂O with adequate leak) and respiratory patterns. Once confirmed, sevoflurane was discontinued. EtSevo concentrations decreased to undetectable levels within approximately 6 minutes, allowing for a smooth extubation without emergence delirium or respiratory complications. No withdrawal symptoms were observed.

## Discussion

CLMs can cause critical airway compromise due to mass effect and significant postoperative swelling following sclerotherapy. Edema typically peaks within 24-72 hours after treatment [6], creating a high-risk period where accidental extubation must be rigorously avoided.

In this case, conventional IV sedatives were insufficient. As detailed in the case presentation, the multi-drug regimen failed to achieve adequate sedation depth, leading to persistent agitation and hemodynamic instability during the first treatment course. A primary limitation of IV agents in this context is the lack of real-time depth monitoring, which often results in unpredictable clinical responses when doses are adjusted. While neuromuscular blockade can ensure immobility, it eliminates spontaneous breathing and significantly increases the risk of catastrophic deterioration should accidental extubation occur in the setting of a distorted airway.

Sevoflurane inhalational sedation provided a safer "middle ground" for this patient. It allowed for rapid titration via EtSevo, achieving stable immobility while preserving spontaneous respiration and reducing the requirement for IV agents. Although evidence on inhalational sedation in the pediatric intensive care unit (PICU) remains limited [1,2], its favorable controllability is a distinct advantage in difficult-airway scenarios. Randomized adult trials [3,4] also demonstrate faster awakening with comparable safety to IV agents, supporting the utility of sevoflurane when traditional sedation strategies fail.

The successful management in this case was further supported by a multidisciplinary extubation evaluation. Combining surgical airway visualization with PICU physiologic readiness enabled safe and controlled timing for extubation.

It is important to note that while the Sedaconda® system is fully approved in Japan, Scandinavia, and most European countries, it has not yet received full FDA approval for general marketing in the United States. However, it has been granted FDA Fast Track designation, and an Early Access Program was approved in April 2025 for specific "difficult-to-sedate" patients. This case contributes to the growing body of evidence supporting its utility in challenging sedation scenarios.

In summary, this case provides several clinical insights. First, it demonstrates the feasibility of sevoflurane rescue sedation for postoperative airway protection in a child with a complex CLM. Second, it shows that stable sedation and predictable emergence can be achieved via EtSevo monitoring, even in difficult-airway scenarios. Third, the use of sevoflurane highlights its utility when multiple IV agents fail to control agitation. Finally, these findings suggest that sevoflurane may serve as a safer intermediary step before considering neuromuscular blockade when immobility is required, but preserving the respiratory drive is paramount.

## Conclusions

Sevoflurane inhalational sedation enabled stable management during the high-risk postoperative swelling phase and ensured a predictable extubation in a pediatric patient with CLM after inadequate IV sedation. This approach proved superior in maintaining hemodynamic stability and controlling agitation while preserving spontaneous ventilation. Inhalational sedation should be considered a valuable option for managing airway-risk pediatric patients in the PICU.
